# Physical Exercise After Solid Organ Transplantation: A Cautionary Tale

**DOI:** 10.3389/ti.2024.12448

**Published:** 2024-02-13

**Authors:** Dimitri Stylemans, Marieke Vandecruys, Sofie Leunis, Sofie Engelborghs, Davide Gargioli, Diethard Monbaliu, Véronique Cornelissen, Amaryllis H. Van Craenenbroeck, Stefan De Smet

**Affiliations:** ^1^ Department of Respiratory Diseases, Pulmonary Rehabilitation, University Hospitals Leuven, Leuven, Belgium; ^2^ Nephrology and Renal Transplantation Research Group, Department of Microbiology, Immunology and Transplantation, KU Leuven, Leuven, Belgium; ^3^ Laboratory of Abdominal Transplantation, Department of Microbiology, Immunology and Transplantation, KU Leuven, Leuven, Belgium; ^4^ Exercise Physiology Research Group, Department of Movement Sciences, KU Leuven, Leuven, Belgium; ^5^ Department of Abdominal Transplant Surgery, University Hospitals Leuven, Leuven, Belgium; ^6^ Transplantoux Foundation, Leuven, Belgium; ^7^ Research Group for Rehabilitation in Internal Disorders, Department of Rehabilitation Sciences, KU Leuven, Leuven, Belgium; ^8^ Department of Nephrology and Kidney Transplantation, University Hospitals Leuven, Leuven, Belgium

**Keywords:** SOT, solid organ transplant, exercise, adverse event, methodological appraisal, critical appraisal, physical activity, side effects

## Abstract

An increasing body of randomized controlled trials suggests the safety of engaging in moderate to vigorous intensity exercise training following solid organ transplantation. Fueled by emerging sport events designed for transplant recipients and the ever-growing body of research highlighting the diverse health benefits of physical activity, transplant recipients are now increasingly participating in strenuous and occasionally competitive physical endeavors that largely surpass those evaluated in controlled research settings. This viewpoint article adopts a cautionary stance to counterbalance the prevalent one-sided optimistic perspective regarding posttransplant physical activity. While discussing methodological limitations, we explore plausible adverse impacts on the cardiovascular, immunological, and musculoskeletal systems. We also examine the physiological consequences of exercising in the heat, at high altitude, and in areas with high air pollution. Risks associated with employing performance-enhancing strategies and the conceivable psychological implications regarding physical activity as a tribute to the ‘gift of life’ are discussed. With a deliberate focus on the potential adverse outcomes of strenuous posttransplant physical activity, this viewpoint aims to restore a balanced dialogue on our comprehension of both beneficial and potentially detrimental outcomes of physical activity that ultimately underscores the imperative of well-informed decision-making and tailored exercise regimens in the realm of posttransplant care.

## Citius Altius, Fortius?

Promoting moderate to vigorous-intensity physical activity is gaining traction as a strategy to address prevalent cardiovascular, metabolic, muscular, and mental comorbidities in solid organ transplant recipients (SOTRs). This is in part supported by direct evidence from controlled intervention studies [1–4] and further driven by strong indirect evidence seen in the general population, translated in the World Health Organization physical activity recommendations [5]. In 2019, a collaborative position statement from the Canadian Society of Transplantation and CAN-RESTORE recommended SOTRs to participate in moderate to vigorous-intensity exercise 3–5 times per week [1]. The absence of sufficient evidence limited the formulation of more specific training recommendations. A surge in initiatives spearheaded by organizations like the World Transplant Games Federation, Transplant Sport, and Transplantoux is now encouraging SOTRs to participate in at times demanding and sometimes competitive physical endeavors. A survey-based investigation involving 220 athletes engaged in the British and World Transplant Games revealed that nearly one-third of respondents aspired to win national and international events, respectively [6]. Notably, over half of the respondents perceived limitations to their performance related to injury, illness, or lack of fitness. Nonetheless, the increasing ambition and potential of SOTRs is evident in successful undertakings such as 130 km cycling races, iron man triathlons, and high-altitude trekking expeditions to for instance the summit of Kilimanjaro [7–10]. Such admirable endeavours are undertaken by a select subpopulation of SOTRs often portrayed as role model. This prompts the question of whether the original Olympic motto ‘*citius, altius, fortius*’ should be embraced within the transplant community. Without contradicting the value of appropriate individualised training programs for SOTRs [1–4] and without discrediting those who have achieved highly competitive goals, this review aims to summarise the current evidence on potential downsides of strenuous physical activity after SOT. This review explores physical activity effects on various organ systems, the influence of climatic conditions, the use of performance-enhancing drugs, and methodologic limitations of the present literature. Occasional speculative arguments will not be avoided, as they can contribute to sparking an open debate that may ultimately lead to improved informed decision-making and implementation of thoughtful, personalised physical activity interventions.

## Organ Systems at Risk

**FIGURE 1:**
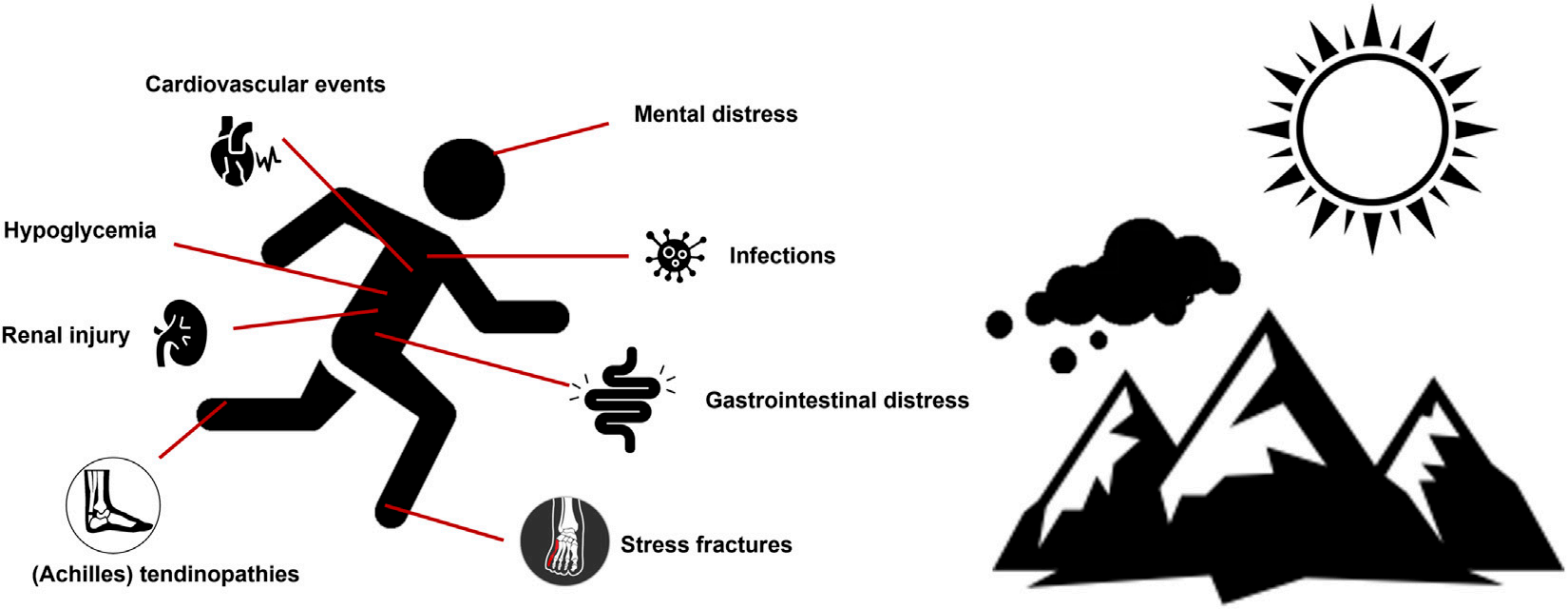
Potential adverse outcomes of participation in posttransplant strenuous physical activity conducted in challenging environmental conditions.

Although poorly researched to date, particularly in SOTRs, strenuous exercise could be postulated to adversely impact various organ systems (Figure 1; Table 1).

**TABLE 1:** Potential adverse effects of strenuous physical exercise in solid organ transplant recipients, and potential strategies to mitigate these adverse effects.

System	Potential adverse effects	Potential mitigation strategies
Musculoskeletal	- Overuse injuries: stress fractures, (Achilles) tendinopathies, and other soft-tissue injuries	- Load management: avoid over- and undertraining
		- Personalized, progressive increase in training volume and intensity
		- Implement resistance training
		- Implement warming-up routines
		- Attention to sufficient recovery (*e.g.,* soft tissue therapy, mobility exercises, active recovery (light exercise), good sleep, cryotherapy, thermotherapy, post-exercise nutrition)
		- Add variety in training routine and limit (the increase in) long duration repetitive movements (*e.g.*, long-distance running)
		- Respect tissue-specific load-capacity ratio
		- Avoid imbalance between energy expenditure and caloric intake
		- In case of overweight: weight loss
		- Training load can be modified by extrinsic factors, such as training surface and footwear
		- Be alert for early symptoms
		- Screen for osteoporosis. When present: calcium-vitamin D supplementation and/or bisphosphonate treatment
Immunological	- Increased risk of infection, in particular that of the upper respiratory and urinary tract	- Avoid overreaching/overtraining
		- Implement infection preventing strategies following strenuous exercise and during strenuous training periods (*e.g.*, hygiene practices, immunization)
		- Prevent dehydration and infrequent voiding
		- Avoid environments that house a high number of pathogens (*e.g.,* natural waters, public swimming pools)
		- Dynamic customization of immunosuppressive regime may be useful, but remains a topic of future research
Cardiovascular	- Cardiovascular events during acute strenuous exercise	- Preparticipation cardiovascular screening (cardiopulmonary exercise test, functional imaging, coronary computed tomography scan, coronary angiography)
	- Increased risk for development of certain cardiovascular diseases (coronary plaques, atrial fibrillation, myocardial fibrosis) in those surpassing the ‘optimal dose of exercise’?	- Personalized, progressive increase in training load
		- Balanced approach regarding long-term training load
Gastrointestinal	- Gastrointestinal distress (*e.g.*, diarrhea, regurgitation, nausea)	- Avoid too strenuous exercise
	- Gut microbial dysbiosis	- Adequate hydration
		- Dietetic measures
Endocrine	- Acute dysregulation of blood glucose levels	- Patient education on exercise-induced modulation of blood glucose and symptom recognition
		- Dietetic and insulin interventions before, during, and after exercise
Renal	- Impaired kidney perfusion	- Avoid too strenuous exercise
	- Acute kidney injury	- Respect muscle load-capacity ratio; prevent excessive exercise-induced muscle damage
	- Rhabdomyolysis	- Maintain good hydration and electrolyte balance
Systemic, renal, cardiovascular, and respiratory systems during exposure to extreme environments	- Acute mountain sickness	- Avoid strenuous exercise in environments characterized by high levels of heat, humidity, altitude, or air pollution
	- Impaired kidney function and kidney injury	- Opt for indoor alternatives
	- Cardiovascular disease	- Heat mitigation strategies: strategically choose time of day, time of year, clothing, hydration, *etc.*
	- Impaired lung function	- Decrease exercise intensity and duration
	- Increased mortality risk	
Psychological	- Mental distress: feelings of guilt or personal failure	- Elicit intrinsic motivation rather than relying on extrinsic factors (*e.g*., leveraging a patient’s feelings of guilt toward their donor) to initiate and/or maintain a physically active lifestyle
Various	- Drug-drug interactions	- Patient education on drug-drug interactions
	- Contaminated supplement use	- Professional counseling regarding medication and nutrition in the context of health and exercise performance
	- High dietary protein intake increasing kidney workload	- Use of high quality batch-tested supplements only, if necessary at all
	- Incorrect use or non-adherence to medication	

### Musculoskeletal Injuries

While osteoporosis is common among patients awaiting transplantation, further bone loss is a typical phenomenon throughout the early posttransplant period [11]. Even patients without a pretransplant history of osteoporosis face an elevated risk of posttransplant osteoporosis and fractures [12, 13]. The incidence of fractures is four to five times higher compared to the general population [14, 15] and can be attributed, in part, to the side effects of immunosuppressive agents such as glucocorticoids, cyclosporine A, and tacrolimus [16–19]. Particularly in individuals engaging in endurance sports, an imbalance between energy expenditure and caloric intake can lead to the relative energy deficiency in sport syndrome, further reducing bone mineral density and increasing the risk for stress fractures [20, 21].

Immunosuppressive agents, together with obesity and/or type 2 diabetes, also increase the risk for tendinopathy [22, 23]. Some evidence indicates heart and kidney transplant recipients to be at elevated risk of (Achilles) tendinopathy, possibly related to fluoroquinolone therapy [24, 25]. Both undertraining and overtraining are known to set healthy athletes at risk for non-contact soft-tissue injury [26]. Tailoring a progressive training load seems essential in injury prevention, particularly in SOTRs already predisposed to injury. Training load not only relates to the volume and intensity of exercise, but also to tissue-specific mechanical stress associated with a given type of exercise. For instance, high-impact sports such as long-distance running amplify the risk of stress fractures in the lower limbs [27, 28]. Vigilance for exercise-induced overuse injuries in SOTRs seems justified. Screening practices with timely bone mineral density measurements and personalized pharmacological and training approaches may be advised. Supported by promising results in heart and lung transplant recipients, the latter may amongst others consist of bisphosphonate treatment, well-planned aerobic weight-bearing exercise, and resistance training [29–31].

### Immune Modulation

Immunosuppressive therapy has turned allotransplantation into a curative option for end-stage organ disease [32]. However, chronic immunosuppressive therapy comes at a price of heightened vulnerability to infections. Especially within the initial year and contingent upon the transplant type, patients are susceptible to common or opportunistic infections, primarily affecting the lungs, gastrointestinal system, and urinary tract [33, 34]. In the general population, engaging in mild to moderate-intensity exercise, *i.e.*, below 60% of peak oxygen uptake (VO_2_peak), promotes immunovigilance and reduces infection incidence [34]. However, a contrasting effect is observed with strenuous exercise, such as prolonged exercise exceeding 60% VO_2_peak [34]. Through complex interactions of transient changes in adaptive and innate immune system components, increased inflammatory responses, and metabolic factors that impair immune cell metabolic capacity, engagement in strenuous exercise leads to temporarily suppressed immune function [35]. In healthy athletes, a typical example of this phenomenon is the six-fold increased risk for upper respiratory tract infection within the first week after a marathon [36]. The interaction with immunosuppressive therapy is less clear. Blood samples from kidney transplant recipients and matched healthy controls, drawn after a strenuous 81-km bicycling trip, showed appositional gene expression upon incubation with bacterial endotoxin lipopolysaccharide [37]. Immune response genes were overrepresented in controls, whereas numerous apoptotic genes were overrepresented in kidney transplant recipients. These findings, albeit in a limited cohort of 10 transplant recipients and 10 healthy controls, suggest that SOTRs are at increased risk for infection upon contact with a pathogen in the early aftermath of strenuous exercise. Note that certain sport environments such as natural waters and public pools house a higher number of pathogens that can lead to illnesses. In addition, endurance sports may predispose individuals to urinary tract infections due to infrequent voiding and dehydration, particularly in women exercising in hot and humid weather conditions [38–40]. One should balance the benefits against potentially unfavorable immunomodulation of strenuous exercise after SOT. Whether dynamic customization of the immunosuppressive regimen is warranted in response to acute or long-term participation in moderate or strenuous exercise awaits examination in future studies.

### Cardiovascular Events

Regular participation in physical activity has been established as a key factor in reducing cardiovascular morbidity and mortality across the general population [5, 41]. However, the cardiovascular advantages of physical activity exhibit a curvilinear dose-response relationship. Surpassing an ‘optimal dose of exercise’ in terms of duration and intensity might raise the potential for coronary plaque development [42–44], atrial fibrillation [45–47], and myocardial fibrosis [48, 49]. Furthermore, during acute high intensity exercise, cardiac workload and blood pressure increase to potentially hazardous levels which may lead to myocardial infarction and sudden cardiac death in susceptible individuals. This is translated in a >100-fold increased risk of acute myocardial infarction in sedentary individuals during participation in vigorous physical activity [50]. This heightened risk is associated with the presence of underlying coronary artery disease. Coronary artery disease is highly prevalent in SOTRs [42, 51–53]; the 5-year cumulative incidence for coronary artery disease after kidney transplant is 7.6% [51], cardiac allograft vasculopathy in heart transplant patients has a prevalence of 18% [52], coronary stenosis is present in one-third of liver transplant candidates [53], and the 5-year cumulative incidence of cardiovascular events after liver transplantation is 14% [54]. Nonetheless, to our knowledge, apart from one study [55], reports of cardiovascular events occurring during physically demanding exercise [7–10] or during controlled exercise-based rehabilitation are lacking. We speculate that this observation is consequent to profound selection bias and in-depth medical preparticipation screening. Some forms of preparticipation screening (i.e., cardiopulmonary exercise test, functional imaging, coronary CT scan and, if necessary, coronary angiography) may indeed be indicated, particularly in those with heightened cardiovascular risk profile planning to engage in moderate and/or vigorous exercise training or physically strenuous endeavors [56].

### Gastrointestinal Distress

Diarrhea is a common issue among SOTRs, irrespective of exercise. Its prevalence varies, ranging from 13% after kidney transplantation to 40% after liver transplantation [57, 58] and may be of infectious origin but is very often related to side effects of immunosuppressive agents such as tacrolimus and mycophenolate [59]. Gut ischemia, together with mechanical (e.g., increasing intra-abdominal pressure) and neuroimmune endocrine factors, is believed to play an important contribution in exercise-induced diarrhea and gastrointestinal distress symptoms such as regurgitation, nausea, and, in some instances, gastrointestinal bleeding [60]. Unspecified non-infectious diarrhea has been associated with elevated risk of graft failure (e.g., dehydration with negative effects on organ perfusion and function), mortality, and gut microbial dysbiosis [61, 62]. Microbial dysbiosis is widely prevalent among transplant recipients and has been associated with mortality [63]. In contrast to moderate-intensity exercise, strenuous exercise of long-duration and/or high intensity can negatively impact intestinal health, as it reduces intestinal blood flow and increases intestinal permeability, leading to impaired gut-barrier function, depressed immune function, and increased risk for viral and bacterial infections [64, 65]. While in the general population evidence suggests that regular moderate-to-vigorous aerobic physical activity reduces the risk of gastrointestinal malignancies [66], diabetes [67], chronic kidney disease [68, 69], fatty liver disease [70], and gut microbial dysbiosis [71], little is known about the impact of strenuous physical exercise on the gastrointestinal tract in SOTRs.

### Diabetes

Approximately 10–40 percent of SOTRs have some form of diabetes mellitus, mainly type 2 diabetes and posttransplant diabetes mellitus [72]. Class I level A recommendations support the increase in weekly physical activity to 150 min of moderate or 75 min of vigorous intensity activity in all patients with type 2 diabetes [73]. The beneficial glucometabolic effects of exercise in SOTRs are remarkably understudied and at least for now lack solid evidence base [74, 75]. Exercise in patients with diabetes treated with insulin or medication improving insulin secretion (e.g., sulfonylurea) requires specific attention. Exercise-induced increase in insulin sensitivity can modulate blood glucose levels till several hours after exercise cessation [76, 77]. In general, long-duration aerobic exercise increases the risk of acute hypoglycemia, but it appears that there is a great intra- and interindividual variation in blood glucose response to a given exercise stimulus [77]. A brief bout of exercise at vigorous intensity on the other hand increases plasma glucose during and briefly after exercise due to a mismatch between gluconeogenesis and muscle glucose utilization [78]. High diabetes prevalence in SOTRs highlights the importance of patient education regarding exercise-induced modulation of blood glucose, hypoglycemia symptoms recognition, blood glucose monitoring, and adequate dietary strategies before, during, and after exercise.

### Renal Injury

Exercise-induced release of noradrenaline during moderate and vigorous-intensity exercise in temperate climate leads to a reduction of kidney blood flow by about 20% and 52%, respectively, [79, 80]. These physiological changes could be postulated to set kidney transplant recipients, many of whom have an estimated glomerular filtration rate below 60 mL/kg/m^2^ [81], at risk for kidney injury. Of course also other transplant groups often suffer compromised kidney function [82–84]. A prospective study of 76 healthy marathon runners reported a 48% incidence of acute renal failure, mostly grade 1 [85]. Based on serum creatinine levels, earlier findings in 23 marathon runners indicated a 55% incidence of acute renal failure, while 74% of participants showed significant increases in tubular biomarkers [86]. Whether these findings truly imply significant kidney injury remains open for debate, but vigilance may be required.

Though a rare condition, muscle breakdown from strenuous exercise may lead to rhabdomyolysis and associated acute kidney failure and liver dysfunction [87]. The risk increases with excessive, high intensity, long duration, eccentric muscle contractions conducted by less trained individuals. Hot environments, electrolyte imbalance, and inadequate protein and carbohydrate intake may further increase the risk [87].

### Environmental Factors

In contrast to training interventions conducted in controlled research environments, real-world settings often involve environmental stressors that were previously unconsidered. These stressors can include factors like heat, humidity, ambient hypoxia (altitude), and air pollution. Extrapolating safety data from research settings in mild environmental conditions or from observational studies involving carefully selected individuals exposed to challenging environments to the wider transplant population in real-world settings is inappropriate.

Exercise in hot and humid climates causes redistribution of cardiac output to the skin. Combined with evaporation-induced dehydration, this may result in an additional 15%–30% reduction in renal blood flow [88–90], a decrease in glomerular filtration rate, and acute tubular injury possibly resulting in acute kidney injury [88–93].

Apart from hot environments, exercise endeavors are not infrequently organized in oxygen-deprived conditions. Acute altitude sickness, ranging from mild to life threatening forms, may develop upon recent ascent to altitudes ≥2,500 m [94]. Transplant recipients have successfully summited high-altitude peaks such as Kilimanjaro (Tanzania). At its peak 5,895 m above sea level, around 80% of healthy sojourners develop acute mountain sickness [95]. The current literature indicates comparable summitting success rates in well-trained transplant recipients compared to healthy controls [8]. Normal physiological responses, including changes in oxygen saturation and heart rate, appear largely similar to those observed in healthy controls when exposed to increasing altitudes [7, 8, 96–98]. However, a higher incidence of arterial hypertension in liver transplant patients has been reported [98], and higher altitude sickness scores have been reported in lung transplant recipients [7]. In the latter, the rise in right ventricular contractility was blunted, indicating impaired cardiac adaptation to hypoxia. The underlying mechanisms behind this phenomenon could be linked to cardiac autonomic dysfunction due to surgical vagal nerve transection in lung transplantation and/or to the neurotoxic effects of immunosuppressive agents like calcineurin inhibitors. High altitude exposure could also have negative impact on the kidneys. Hypoxia can trigger the development of acute and chronic kidney failure [99, 100]. Acute hypoxia at high-altitude triggers hyperventilation with subsequent development of respiratory alkalosis, for which the kidneys need to compensate [100]. It also increases the renal excretion of sodium and water, potentially decreasing renal perfusion with subsequent reductions in glomerular filtration [100]. High altitude may also lead to high-altitude renal syndrome in which a combination of polycythemia, hyperuricemia, hypertension, and proteinuria coexist and can induce nephropathy with different histopathological features such as glomerular hypertrophy, basement membrane thickening, glomerulosclerosis, and fibrosis [101]. Hypoxia is also known to play a key role in the progression of chronic kidney disease to end stage kidney disease [100].

Young physically inactive adults residing in regions characterized by high levels of particulate matter, commonly encountered in big cities and regions with high levels of air pollution, exhibit an augmented susceptibility to cardiovascular disease upon elevating their physical activity levels to ≥1,000 MET-min/week (equivalent to approximately ≥4 h of moderate-intensity physical activity) [102]. Furthermore, engagement in vigorous but not moderate-intensity physical activity in areas with high levels of air pollution appears to escalate mortality risks among older adults [103]. These findings hold particular relevance to lung transplant candidates and recipients, as poor air quality in their living environments correlate with adverse waitlist occurrences and compromised lung function, respectively [104, 105]. Consequently, exercise recommendations must factor in air pollution levels, emphasizing that, in areas with poor air quality, vigorous-intensity exercise should be conducted indoors or substituted with moderate-intensity alternatives.

### Mental Distress—The “Gift of Life” Metaphor

Receiving solid organ transplantation and thereby the “gift of life” may induce substantial psychological distress among recipients [106]. Upon receiving a donor organ, recipients may experience a sense of obligation not only towards their donors, but also towards the medical team and transplant community [107]. It is not uncommon practice among healthcare practitioners to leverage the “gift of life” metaphor as a potent means of fostering motivation towards adopting a healthy physically active lifestyle. “It is a minimal gesture to honor your donor.” Therefore, inability to commence or sustain suitable levels of posttransplant physical activity, regardless of the causes, can evoke sentiments of guilt and personal failure. Research in 134 kidney transplant patients indicated the presence of feeling of guilt in the large majority of patients, with an average guilt score of 69 on a Visual Analogue Scale from 0-100 [108]. It is important to recognize the potential unintended mental strain that patients might undergo and to persist in viewing them as individuals with distinct needs and experiences. Upholding patients’ autonomy and fostering shared decision-making could offer a more ethical and sustainable strategy for interventions targeting the enhancement of patients’ physical activity behavior.

## Performance Enhancers: Caveats

In situations where performance enhancement is pursued, the use of performance enhancing strategies lurks around the corner. Notably, for pain prevention or relief, a significant portion (12%–48%) of healthy participants in endurance sport events have been reported to utilize non-steroidal anti-inflammatory drugs or analgesics [109–111]. Non-steroidal anti-inflammatory drugs have been shown to exert toxicity on the kidneys and gastrointestinal tract and to promote arterial hypertension [112]. Furthermore, these drugs can potentially alter blood concentrations of immunosuppressive medications through drug-drug interactions [113–115]. The same concern exists regarding other over-the-counter drugs and dietary supplements. Supplement manufacturers are not obligated to conduct third-party testing of their product’s safety, efficacy, or potential contamination by substances such as anabolic androgenic steroids or stimulants [116]. Such contamination can arise either inadvertently due to substandard manufacturing practices or deliberately with the aim of enhancing product efficacy [117]. The prevalence of supplement use within athletic populations varies widely, spanning from 11% to 100%, depending upon factors such as the definition used to call a product a supplement, the timeframe considered, and the data collection methodology in the different studies [118]. Apart from carbohydrate and protein supplements, athletes predominantly turn to minerals and vitamins in their endeavors to enhance performance, promote health, and aid recovery [118]. Nutritional supplement adoption is similarly widespread among SOTRs. A study involving liver, kidney, and combined lung-heart transplant recipients revealed that 58% of the patients consistently integrated supplements into their regimens, independent of exercise participation, encompassing vitamins, minerals, and diverse herbs asserted to possess therapeutic advantages [119]. A notable concern arises from the fact that in roughly 75% of cases, the treating physician remained uninformed about patients’ supplement intake.

Transplant recipients should be cautious when mimicking the dietary habits of professional athletes. For example, the International Association of Athletics Federations recommends a daily protein intake of 1.3–1.7 g/kg for weight-stable athletes, and 1.6–2.4 g/kg for athletes aiming to attain weight loss while preserving lean body mass [120]. This stands in contrast to the existing daily dietary protein recommendations for kidney transplant recipients, which are currently established at 0.8 g/kg of body weight [121, 122]. Substantial dietary protein intake following kidney transplantation has been hypothesized to contribute to elevated blood pressure, secondary graft failure, and an increased risk of cardiovascular events [121, 123, 124].

Lastly, competitive and performance-oriented transplant recipients may be inclined to temporarily adjust their medication schedules in order to achieve performance advantages. Chronotropic incompetence induced by beta blockers can reduce VO_2_peak by 5%–15%, significantly diminishing cardiorespiratory exercise performance [125]. Patients participating in challenging aerobic events could thus be tempted to temporarily decrease the dosage of these ergolytic agents. This is of course inadvisable, as this medication provides a significant survival benefit in certain conditions such as heart failure or after acute myocardial infarction [126]. A similar assumption could apply for the inappropriate increase in erythropoietin use to achieve above normal total hemoglobin mass in patients receiving erythropoietin treatment, e.g., about one in ten kidney transplant patients [127].

## A Critical Appraisal of the Research Methodology in the Field of Exercise in SOTRs

**FIGURE 2:**
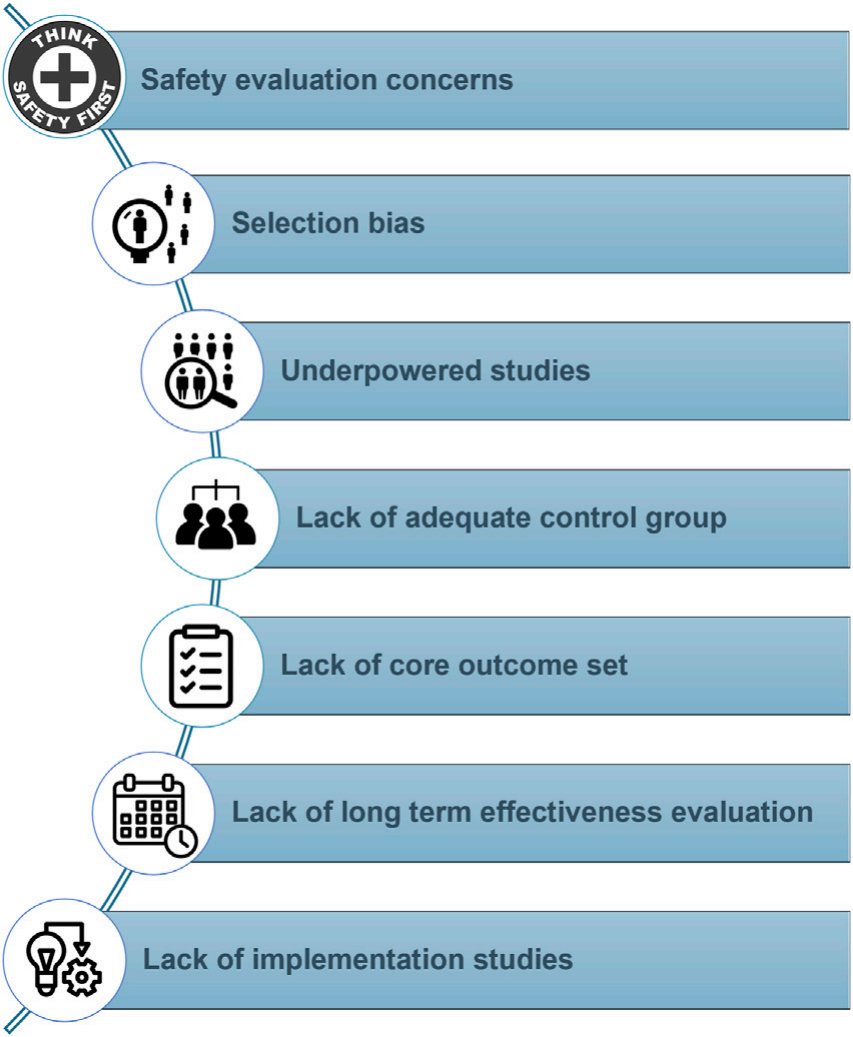
Common methodological concerns present in the current literature regarding physical activity and exercise interventions after solid organ transplantation.

Various randomized controlled trials (RCTs) suggest that safety concerns are not readily apparent in posttransplant exercise-based rehabilitation [74, 75, 128]. However, RCTs and systematic reviews of RCTs are commonly misconstrued as the primary sources for assessing adverse intervention effects. Apart from the limited data the present literature enables us to make strong safety claims, other methodological concerns are present that limit statements about training intervention’s effectiveness, generalization, and implementation (Figure 2). A recent Cochrane review that evaluated the harms and benefits of exercise interventions in liver transplant recipients corroborates our critical appraisal [4]. The authors concluded that there were very few data on adverse events outcomes, and that based on very low-certainty evidence the role of exercise training in affecting mortality, health-related quality of life, and physical function was very uncertain [4].

### Safety Evaluation Concerns

In the realm of posttransplant exercise training, RCTs tend to prioritize intervention benefits, while the evaluation of harms is either demoted to a secondary role or omitted altogether. A recent systematic review of RCTs examining exercise training after SOT showed that adverse events were explicitly documented in merely eight out of the 21 studies encompassed [128]. Additionally, safety assessment frequently relied on retrospective self-reporting of adverse events. This approach might have resulted in the omission of harms that patients deemed insufficiently severe or significant, as well as harms that they did not perceive to be connected to the study. Although we did not perform a systematic literature search to confirm our hypothesis, it is likely that training studies in SOTRs, in line with similar studies in SOT candidates [129], not only poorly define and describe safety but also fail to inform the reader whether dropouts may have been related to adverse events. Future studies should *a priori* define adverse events, include a prospective evaluation of potential harms, and clearly describe whether dropouts could potentially have been related to the applied intervention. Safety claims based on RCTs have additional shortcomings in that most of these studies are underpowered and of insufficient duration to detect anything beyond the most common harms.

### Selection Bias and Underpowered Studies

Strict eligibility criteria and substantial selection bias considerably limit the generalizability of the RCTs’ conclusions on harms and benefits and lead to underpowered studies. Selection bias is a significant concern in the present research field. It results in a sample that is not representative of the transplant population in real-world settings. A recent review on RCTs showed that only 35% of the approached kidney transplant recipients were found eligible and willing to participate in exercise RCTs [74]. In liver transplant recipients, only one in three approached patients were included in the final analyses [75]. Systematic reviews in heart and lung transplant recipients have reported difficulties in evaluating selection bias due to issues with the selection procedures in a significant number of analyzed studies [2, 3]. Future literature reviews on safety of exercise after transplantation should also include other types of studies on top of RCTs, given that observational studies may be less restrictive in their eligibility criteria and achieve larger sample sizes [130, 131].

### Lack of Adequate Control Group and Core Outcome Set

The absence of blinding constitutes another prevalent bias in exercise RCTs. Given the nature of the intervention, blinding participants to the exercise regimen is essentially unattainable, rendering the intervention arm susceptible to expectancy effects [132]. Consequently, to facilitate more credible between-group comparisons of intervention effects, it is recommended to employ an attention control group or a sham exercise comparator (e.g., flexibility exercise training) instead of usual care. Furthermore, the lack of a standardized set of core outcomes evaluated throughout the different studies, great heterogeneity in the applied training interventions, and other methodological limitations make it difficult to draw strong conclusions and recommendations from the existing literature.

### Lack of Long-Term Behavior Change and Effectiveness Evaluation

Lack of long-term follow-up is a major shortcoming, as it is required to evaluate long-term effects on hard objective outcomes such as mortality, graft function, and the incidence of cardiovascular or other life-threatening events [1, 3, 74, 75]. While exercise interventions have demonstrated favorable outcomes concerning cardiorespiratory fitness, muscle strength, and quality of life in the short term, these advantages are transient and do not last over the long term [132–138]. Short-term exercise-based rehabilitation after transplantation may be recommended for specific patients to enhance their physical and psychological capacity for physical activity. However, continuous engagement in lifelong exercise training is likely unfeasible for the majority of the population [133]. Consequently, in our perspective, exercise interventions should consistently be preceded by tailored physical activity behavioral interventions when aiming for long-term clinical impact [139].

### Lack of Implementation Studies

Despite the multifaced health benefits associated with exercise RCTs, the implementation of such interventions remains challenging. The majority of exercise studies have been conducted under strict research conditions, often neglecting critical aspects such as stakeholder involvement throughout the whole lifespan of a research project; *i.e.*, the co-design of the intervention, public and patient involvement in the recruitment strategy, selection of relevant outcomes, interpretation, and dissemination of study results. Moreover, the local context in which the intervention is delivered, and the development of tailored implementation strategies adapted to that context have also been overlooked. In addition to evaluating the efficacy and effectiveness of exercise interventions, the study of methods that promote systematic uptake of these interventions in clinical practice may offer valuable insights with real-world impact [140]. Therefore, there is a critical need to bridge the gap between research and practice and integrate exercise and physical activity interventions into routine clinical care to promote their implementation and ultimately improve patient outcomes.

## The Difference Between Medicine and Poison Lies in the Dose

In this review, we delved into the existing literature to analyse the impact of (strenuous) physical activity on SOTRs. Embracing a stance akin to the devil’s advocate, we did not avoid sometimes speculative arguments hoping to stimulate dialogue within the posttransplant exercise research community. It is crucial to emphasize that while exercise can serve as a potent therapeutic intervention after transplantation and is probably underutilized in the transplant population, the line between its medicinal benefits and potential harm lies in the dosage administered. Tailoring exercise frequency, intensity, duration, and type to the unique needs of each individual as well as continuous monitoring for potential adverse events is imperative. Although past case reports suggest the feasibility of completing tremendous physical endeavours after transplantation, a one-size-fits-all approach is not viable, given the associated risks spanning various organ systems, including the transplanted organ. Appropriate precautions may sometimes be advised to mitigate potential adverse events. Lastly, we underscored frequent methodological concerns in the present research field as a call for more high-quality studies.

## Abbreviations

SOT, solid organ transplant; SOTRs, solid organ transplant recipients; RCTs, randomized controlled trials; VO_2_peak, peak oxygen uptake.

## References

[1] Janaudis-Ferreira T Mathur S Deliva R Howes N Patterson C Räkel A Exercise for Solid Organ Transplant Candidates and Recipients: A Joint Position Statement of the Canadian Society of Transplantation and CAN-RESTORE. Transplantation (2019) 103(9):e220–38. 10.1097/TP.0000000000002806 31461743

[2] Gutierrez-Arias R Martinez-Zapata MJ Gaete-Mahn MC Osorio D Bustos L Melo Tanner J Exercise Training for Adult Lung Transplant Recipients. Cochrane Database Syst Rev (2021) 7(7):CD012307. 10.1002/14651858.CD012307.pub2 34282853 PMC8406964

[3] Anderson L Nguyen TT Dall CH Burgess L Bridges C Taylor RS. Exercise-Based Cardiac Rehabilitation in Heart Transplant Recipients. Cochrane Database Syst Rev (2017) 4(4):CD012264. 10.1002/14651858.CD012264.pub2 28375548 PMC6478176

[4] Pérez-Amate È Roqué-Figuls M Fernández-González M Giné-Garriga M. Exercise Interventions for Adults After Liver Transplantation. Cochrane database Syst Rev (2023) 5(5):CD013204. 10.1002/14651858.CD013204.pub2 37204002 PMC10201528

[5] Bull FC Al-Ansari SS Biddle S Borodulin K Buman MP Cardon G World Health Organization 2020 Guidelines on Physical Activity and Sedentary Behaviour. Br J Sports Med (2020) 54(24):1451–62. 10.1136/bjsports-2020-102955 33239350 PMC7719906

[6] Hames T Leddington-Wright S Thake CD Price M. Characteristics of Transplant Athletes Competing at National and International Transplant Games. BMJ Open Sport Exerc Med (2022) 8(1):e001248. 10.1136/bmjsem-2021-001248 PMC8886416 35309375

[7] Schrutka L Slama A Muehlbacher J Bessa V Lichtenegger P Ghimessy Á Cardiopulmonary Response to High-Altitude Mountaineering in Lung Transplant Recipients-The Jebel Toubkal Experience. Scand J Med Sci Sports (2021) 31(10):1941–8. 10.1111/sms.14008 34170580

[8] van Adrichem EJ Siebelink MJ Rottier BL Dilling JM Kuiken G van der Schans CP Tolerance of Organ Transplant Recipients to Physical Activity During a High-Altitude Expedition: Climbing Mount Kilimanjaro. PLoS One (2015) 10(11):e0142641. 10.1371/journal.pone.0142641 26606048 PMC4659574

[9] Haykowsky MJ Riess KJ Schneider CA. Ironman Triathlon Performance Pre- and Post-Heart Transplant. J Heart Lung Transpl (2015) 34(5):756. 10.1016/j.healun.2014.12.015 25727770

[10] Cappuccilli M Mosconi G Roi GS De Fabritiis M Totti V Merni F Inflammatory and Adipose Response in Solid Organ Transplant Recipients After a Marathon Cycling Race. Transplant. Proc. (2016) 48(2):408–14. 27109967 10.1016/j.transproceed.2016.02.001

[11] Anastasilakis AD Tsourdi E Makras P Polyzos SA Meier C McCloskey EV Bone Disease Following Solid Organ Transplantation: A Narrative Review and Recommendations for Management From the European Calcified Tissue Society. Bone (2019) 127:401–18. 10.1016/j.bone.2019.07.006 31299385

[12] Chen H Lai YR Yang Y Gau SY Huang CY Tsai TH High Risk of Osteoporosis and Fracture Following Solid Organ Transplantation: A Population-Based Study. Front Endocrinol (2023) 14:1167574. 10.3389/fendo.2023.1167574 PMC10242127 37288297

[13] Guichelaar MM Schmoll J Malinchoc M Hay JE. Fractures and Avascular Necrosis Before and After Orthotopic Liver Transplantation: Long-Term Follow-Up and Predictive Factors. Hepatology (2007) 46(4):1198–207. 10.1002/hep.21805 17654700

[14] Nikkel LE Hollenbeak CS Fox EJ Uemura T Ghahramani N. Risk of Fractures After Renal Transplantation in the United States. Transplantation (2009) 87(12):1846–51. 10.1097/TP.0b013e3181a6bbda 19543063

[15] Lim WH Ng CH Ow ZGW Ho OTW Tay PWL Wong KL A Systematic Review and Meta-Analysis on the Incidence of Osteoporosis and Fractures After Liver Transplant. Transpl Int (2021) 34(6):1032–43. 10.1111/tri.13863 33835638

[16] Cohen A Shane E. Osteoporosis After Solid Organ and Bone Marrow Transplantation. Osteoporos Int (2003) 14(8):617–30. 10.1007/s00198-003-1426-z 12908095

[17] Edwards BJ Desai A Tsai J Du H Edwards GR Bunta AD Elevated Incidence of Fractures in Solid-Organ Transplant Recipients on Glucocorticoid-Sparing Immunosuppressive Regimens. J Osteoporos (2011) 2011:591793. 10.4061/2011/591793 21922049 PMC3172972

[18] Cunningham J. Posttransplantation Bone Disease. Transplantation (2005) 79(6):629–34. 10.1097/01.tp.0000149698.79739.ef 15785362

[19] Bellorin-Font E Rojas E Martin KJ. Bone Disease in Chronic Kidney Disease and Kidney Transplant. Nutrients (2022) 15(1):167. 10.3390/nu15010167 36615824 PMC9824497

[20] Matcuk GR Jr Mahanty SR Skalski MR Patel DB White EA Gottsegen CJ. Stress Fractures: Pathophysiology, Clinical Presentation, Imaging Features, and Treatment Options. Emerg Radiol (2016) 23(4):365–75. 10.1007/s10140-016-1390-5 27002328

[21] Cabre HE Moore SR Smith-Ryan AE Hackney AC. Relative Energy Deficiency in Sport (RED-S): Scientific, Clinical, and Practical Implications for the Female Athlete. Dtsch Z Sportmed (2022) 73(7):225–34. 10.5960/dzsm.2022.546 36479178 PMC9724109

[22] Millar NL Silbernagel KG Thorborg K Kirwan PD Galatz LM Abrams GD Tendinopathy. Nat Rev Dis Primers (2021) 7(1):1. 10.1038/s41572-020-00234-1 33414454

[23] Humbyrd CJ Bae S Kucirka LM Segev DL. Incidence, Risk Factors, and Treatment of Achilles Tendon Rupture in Patients With End-Stage Renal Disease. Foot Ankle Int (2018) 39(7):821–8. 10.1177/1071100718762089 29582683 PMC6023765

[24] Donck JB Segaert MF Vanrenterghem YF. Fluoroquinolones and Achilles Tendinopathy in Renal Transplant Recipients. Transplantation (1994) 58(6):736–7. 10.1097/00007890-199409270-00021 7940700

[25] Barge-Caballero E Crespo-Leiro MG Paniagua-Martín MJ Muñiz J Naya C Bouzas-Mosquera A Quinolone-Related Achilles Tendinopathy in Heart Transplant Patients: Incidence and Risk Factors. J Heart Lung Transpl (2008) 27(1):46–51. 10.1016/j.healun.2007.09.021 18187086

[26] Gabbett TJ. The Training—Injury Prevention Paradox: Should Athletes Be Training Smarter and Harder? Br J Sports Med (2016) 50:273–80. 10.1136/bjsports-2015-095788 26758673 PMC4789704

[27] Fredericson M Jennings F Beaulieu C Matheson GO. Stress Fractures in Athletes. Top Magn Reson Imaging (2006) 17(5):309–25. 10.1097/RMR.0b013e3180421c8c 17414993

[28] Scheer V Tiller NB Doutreleau S Khodaee M Knechtle B Pasternak A Potential Long-Term Health Problems Associated With Ultra-Endurance Running: A Narrative Review. Sports Med (2022) 52(4):725–40. 10.1007/s40279-021-01561-3 34542868 PMC8450723

[29] Lems WF Paccou J Zhang J Fuggle NR Chandran M Harvey NC Vertebral Fracture: Epidemiology, Impact and Use of DXA Vertebral Fracture Assessment in Fracture Liaison Services. Osteoporosis (2021) 32(3):399–411. 10.1007/s00198-020-05804-3 PMC7929949 33475820

[30] Stein EM Ortiz D Jin Z McMahon DJ Shane E. Prevention of Fractures After Solid Organ Transplantation: A Meta-Analysis. J Clin Endocrinol Metab (2011) 96(11):3457–65. 10.1210/jc.2011-1448 21849532 PMC3205901

[31] Benedetti MG Furlini G Zati A Letizia Mauro G. The Effectiveness of Physical Exercise on Bone Density in Osteoporotic Patients. Biomed Res Int (2018) 2018:4840531. 10.1155/2018/4840531 30671455 PMC6323511

[32] Timsit JF Sonneville R Kalil AC Bassetti M Ferrer R Jaber S Diagnostic and Therapeutic Approach to Infectious Diseases in Solid Organ Transplant Recipients. Intensive Care Med (2019) 45(5):573–91. 10.1007/s00134-019-05597-y 30911807 PMC7079836

[33] Singh N Limaye AP. Infections in Solid-Organ Transplant Recipients. In: Mandell, Douglas, and Bennett's Principles and Practice of Infectious Diseases. Amsterdam, Netherlands: Elesiver (2015). p. 3440–52. 10.1016/B978-1-4557-4801-3.00313-1

[34] Nieman DC Wentz LM. The Compelling Link Between Physical Activity and the Body's Defense System. J Sport Health Sci (2019) 8(3):201–17. 10.1016/j.jshs.2018.09.009 31193280 PMC6523821

[35] Nieman DC Gillitt ND Sha W. Identification of a Select Metabolite Panel for Measuring Metabolic Perturbation in Response to Heavy Exertion. Metabolomics (2018) 14:147. 10.1007/s11306-018-1444-7 30830401

[36] Nieman DC Johanssen LM Lee JW Arabatzis K. Infectious Episodes in Runners Before and After the Los Angeles Marathon. J Sports Med Phys Fitness (1990) 30(3):316–28. 2266764

[37] Königsrainer I Löffler M Bühler S Walter M Schafbuch L Beckert S Impact of Endotoxin Exposure After Exhausting Exercise on the Immune System in Solid Organ Transplant Recipients. Exerc Immunol Rev (2012) 18:177–83. 22876728

[38] Beetz R. Mild Dehydration: A Risk Factor of Urinary Tract Infection? Eur J Clin Nutr (2003) 57(Suppl. 2):S52–8. 10.1038/sj.ejcn.1601902 14681714

[39] Simmering JE Polgreen LA Cavanaugh JE Erickson BA Suneja M Polgreen PM. Warmer Weather and the Risk of Urinary Tract Infections in Women. J Urol (2021) 205(2):500–6. 10.1097/JU.0000000000001383 32945727 PMC8477900

[40] Gaither TW Awad MA Murphy GP Metzler I Sanford T Eisenberg ML Cycling and Female Sexual and Urinary Function: Results From a Large, Multinational, Cross-Sectional Study. J Sex Med (2018) 15(4):510–8. 10.1016/j.jsxm.2018.02.004 29548713

[41] Lear SA Hu W Rangarajan S Gasevic D Leong D Iqbal R The Effect of Physical Activity on Mortality and Cardiovascular Disease in 130 000 People From 17 High-Income, Middle-Income, and Low-Income Countries: The PURE Study. Lancet (2017) 390(10113):2643–54. 10.1016/S0140-6736(17)31634-3 28943267

[42] Franklin BA. Preventing Exercise-Related Cardiovascular Events: Is a Medical Examination More Urgent for Physical Activity or Inactivity? Circulation (2014) 129(10):1081–4. 10.1161/CIRCULATIONAHA.114.007641 24421369

[43] Eijsvogels TMH Thompson PD Franklin BA. The "Extreme Exercise Hypothesis": Recent Findings and Cardiovascular Health Implications. Curr Treat Options Cardiovasc Med (2018) 20(10):84. 10.1007/s11936-018-0674-3 30155804 PMC6132728

[44] De Bosscher R Dausin C Claus P Bogaert J Dymarkowski S Goetschalckx K Lifelong Endurance Exercise and Its Relation With Coronary Atherosclerosis. Eur Heart J (2023) 44(26):2388–99. 10.1093/eurheartj/ehad152 36881712 PMC10327878

[45] Abdulla J Nielsen JR. Is the Risk of Atrial Fibrillation Higher in Athletes Than in the General Population? A Systematic Review and Meta-Analysis. Europace (2009) 11(9):1156–9. 10.1093/europace/eup197 19633305

[46] Mont L Elosua R Brugada J. Endurance Sport Practice as a Risk Factor for Atrial Fibrillation and Atrial Flutter. Europace (2009) 11(1):11–7. 10.1093/europace/eun289 18988654 PMC2638655

[47] Calvo N Ramos P Montserrat S Guasch E Coll-Vinent B Domenech M Emerging Risk Factors and the Dose-Response Relationship Between Physical Activity and Lone Atrial Fibrillation: AProspective Case-Control Study. Europace (2016) 18(1):57–63. 10.1093/europace/euv216 26333377 PMC4739323

[48] Wilson M O’Hanlon R Prasad S Deighan A Macmillan P Oxborough D Diverse Patterns of Myocardial Fibrosis in Lifelong, Veteran Endurance Athletes. J Appl Physiol (2011) 110(6):1622–6. 10.1152/japplphysiol.01280.2010 21330616 PMC3119133

[49] van de Schoor FR Aengevaeren VL Hopman MTE Oxborough DL George KP Thompson PD Myocardial Fibrosis in Athletes. Mayo Clin Proc (2016) 91:1617–31. 10.1016/j.mayocp.2016.07.012 27720455

[50] Franklin BA Thompson PD Al-Zaiti SS Albert CM Hivert MF Levine BD Exercise-Related Acute Cardiovascular Events and Potential Deleterious Adaptations Following Long-Term Exercise Training: Placing the Risks Into Perspective-An Update: A Scientific Statement From the American Heart Association. Circulation (2020) 141(13):e705–e736. 10.1161/CIR.0000000000000749 32100573

[51] Israni AK Snyder JJ Skeans MA Peng Y Maclean JR Weinhandl ED Predicting Coronary Heart Disease After Kidney Transplantation: Patient Outcomes in Renal Transplantation (PORT) Study. Am J Transpl (2010) 10(2):338–53. 10.1111/j.1600-6143.2009.02949.x 20415903

[52] Prada-Delgado O Estévez-Loureiro R Paniagua-Martín MJ López-Sainz A Crespo-Leiro MG. Prevalence and Prognostic Value of Cardiac Allograft Vasculopathy 1 Year After Heart Transplantation According to the ISHLT Recommended Nomenclature. J Heart Lung Transpl (2012) 31(3):332–3. 10.1016/j.healun.2011.12.006 22333404

[53] Fussner LA Heimbach JK Fan C Dierkhising R Coss E Leise MD Cardiovascular Disease After Liver Transplantation: When, What, and Who Is at Risk. Liver Transpl (2015) 21(7):889–96. 10.1002/lt.24137 25880971

[54] D'Avola D Cuervas-Mons V Martí J Ortiz de Urbina J Lladó L Jimenez C Cardiovascular Morbidity and Mortality After Liver Transplantation: The Protective Role of Mycophenolate Mofetil. Liver Transplant (2017) 23(4):498–509. 10.1002/lt.24738 28160394

[55] Nytrøen K Rustad LA Aukrust P Ueland T Hallén J Holm I High-Intensity Interval Training Improves Peak Oxygen Uptake and Muscular Exercise Capacity in Heart Transplant Recipients. Am J Transplant (2012) 12(11):3134–42. 10.1111/j.1600-6143.2012.04221.x 22900793

[56] Pelliccia A Sharma S Gati S Bäck M Börjesson M Caselli S 2020 ESC Guidelines on Sports Cardiology and Exercise in Patients With Cardiovascular Disease. Eur Heart J (2020) 42(1):17–96. 10.1093/eurheartj/ehaa605 32860412

[57] Gil-Vernet S Amado A Ortega F Alarcón A Bernal G Capdevila L Gastrointestinal Complications in Renal Transplant Recipients: MITOS Study. Transplant Proc (2007) 39(7):2190–3. 10.1016/j.transproceed.2007.07.015 17889134

[58] Herrero JI Benlloch S Bernardos A Bilbao I Castells L Castroagudin JF Gastrointestinal Complications in Liver Transplant Recipients: MITOS Study. Transplant Proc (2007) 39(7):2311–3. 10.1016/j.transproceed.2007.06.012 17889174

[59] Angarone M Snydman DR, AST ID Community of Practice. Diagnosis and Management of Diarrhea in Solid-Organ Transplant Recipients: Guidelines From the American Society of Transplantation Infectious Diseases Community of Practice. Clin Transpl (2019) 33(9):e13550. 10.1111/ctr.13550 30913334

[60] de Oliveira EP Burini RC. The Impact of Physical Exercise on the Gastrointestinal Tract. Curr Opin Clin Nutr Metab Care (2009) 12(5):533–8. 10.1097/MCO.0b013e32832e6776 19535976

[61] Bunnapradist S Neri L Wong W Lentine KL Burroughs TE Pinsky BW Incidence and Risk Factors for Diarrhea Following Kidney Transplantation and Association With Graft Loss and Mortality. Am J Kidney Dis (2008) 51(3):478–86. 10.1053/j.ajkd.2007.11.013 18295064

[62] Lee JR Magruder M Zhang L Westblade LF Satlin MJ Robertson A Gut Microbiota Dysbiosis and Diarrhea in Kidney Transplant Recipients. Am J Transplant (2019) 19(2):488–500. 10.1111/ajt.14974 29920927 PMC6301138

[63] Swarte JC Li Y Hu S Björk JR Gacesa R Vich Vila A Gut Microbiome Dysbiosis Is Associated With Increased Mortality After Solid Organ Transplantation. Sci Translational Med (2022) 14(660):eabn7566. 10.1126/scitranslmed.abn7566 36044594

[64] Wosinska L Cotter PD O'Sullivan O Guinane C. The Potential Impact of Probiotics on the Gut Microbiome of Athletes. Nutrients (2019) 11(10):2270. 10.3390/nu11102270 31546638 PMC6835687

[65] Wegierska AE Charitos IA Topi S Potenza MA Montagnani M Santacroce L. The Connection Between Physical Exercise and Gut Microbiota: Implications for Competitive Sports Athletes. Sports Med (2022) 52(10):2355–69. 10.1007/s40279-022-01696-x 35596883 PMC9474385

[66] Rock CL Thomson C Gansler T Gapstur SM McCullough ML Patel AV American Cancer Society Guideline for Diet and Physical Activity for Cancer Prevention. CA: Cancer J Clinicians (2020) 70(4):245–71. 10.3322/caac.21591 32515498

[67] Kanaley JA Colberg SR Corcoran MH Malin SK Rodriguez NR Crespo CJ Exercise/Physical Activity in Individuals With Type 2 Diabetes: A Consensus Statement From the American College of Sports Medicine. Med Sci Sports Exerc (2022) 54(2):353–68. 10.1249/MSS.0000000000002800 35029593 PMC8802999

[68] Ma Q Gao Y Lu J Liu X Wang R Shi Y The Effect of Regular Aerobic Exercise on Renal Function in Patients With CKD: A Systematic Review and Meta-Analysis. Front Physiol (2022) 13:901164. 10.3389/fphys.2022.901164 36225309 PMC9549134

[69] Kelly JT Su G Zhang L Qin X Marshall S González-Ortiz A Modifiable Lifestyle Factors for Primary Prevention of CKD: A Systematic Review and Meta-Analysis. J Am Soc Nephrol (2021) 32(1):239–53. 10.1681/ASN.2020030384 32868398 PMC7894668

[70] Younossi ZM Zelber-Sagi S Henry L Gerber LH. Lifestyle Interventions in Nonalcoholic Fatty Liver Disease. Nat Rev Gastroenterol Hepatol (2023) 20:708–22. 10.1038/s41575-023-00800-4 37402873

[71] Clauss M Gérard P Mosca A Leclerc M. Interplay Between Exercise and Gut Microbiome in the Context of Human Health and Performance. Front Nutr (2021) 8:637010. 10.3389/fnut.2021.637010 34179053 PMC8222532

[72] Dos Santos Q Hornum M Terrones-Campos C Crone CG Wareham NE Soeborg A Posttransplantation Diabetes Mellitus Among Solid Organ Recipients in a Danish Cohort. Transpl Int (2022) 35:10352. 10.3389/ti.2022.10352 35449717 PMC9016119

[73] Marx N Federici M Schütt K Müller-Wieland D Ajjan RA Antunes MJ 2023 ESC Guidelines for the Management of Cardiovascular Disease in Patients With Diabetes. Eur Heart J (2023) 44(39):4043–140. 10.1093/eurheartj/ehad192 37622663

[74] De Smet S Van Craenenbroeck AH. Exercise Training in Patients After Kidney Transplantation. Clin Kidney J (2021) 14(Suppl. 2):ii15–ii24. 10.1093/ckj/sfab022 33981416 PMC8101622

[75] De Smet S O'Donoghue K Lormans M Monbaliu D Pengel L. Does Exercise Training Improve Physical Fitness and Health in Adult Liver Transplant Recipients? A Systematic Review and Meta-Analysis. Transplantation (2023) 107(1):e11–e26. 10.1097/TP.0000000000004313 36192838

[76] Colberg SR Sigal RJ Yardley JE Riddell MC Dunstan DW Dempsey PC Physical Activity/Exercise and Diabetes: A Position Statement of the American Diabetes Association. Diabetes Care (2016) 39(11):2065–79. 10.2337/dc16-1728 27926890 PMC6908414

[77] Tyler NS Mosquera-Lopez C Young GM El Youssef J Castle JR Jacobs PG. Quantifying the Impact of Physical Activity on Future Glucose Trends Using Machine Learning. iScience (2022) 25(3):103888. 10.1016/j.isci.2022.103888 35252806 PMC8889374

[78] Adams OP. The Impact of Brief High-Intensity Exercise on Blood Glucose Levels. Diabetes Metab Syndr Obes Targets Ther (2013) 6:113–22. 10.2147/DMSO.S29222 PMC3587394 23467903

[79] DiBona GF. Physiology in Perspective: The Wisdom of the Body. Neural Control of the Kidney. Am J Physiol Regul Integr Comp Physiol (2005) 289(3):R633–41. 10.1152/ajpregu.00258.2005 16105818

[80] Kotoku K Yasuno T Kawakami S Fujimi K Matsuda T Nakashima S Effect of Exercise Intensity on Renal Blood Flow in Patients With Chronic Kidney Disease Stage 2. Clin Exp Nephrol (2019) 23(5):621–8. 10.1007/s10157-018-01685-3 30729347

[81] Kasiske BL Israni AK Snyder JJ Skeans MA. The Relationship Between Kidney Function and Long-Term Graft Survival After Kidney Transplant. Am J Kidney Dis (2011) 57(3):466–75. 10.1053/j.ajkd.2010.10.054 21257243

[82] López-López I Robles López AI Del Prado JMA Benot AR Cabrera SS Morales MLA. Prevalence of Chronic Kidney Disease After Heart Transplant: A Single Center Experience. Transplant Proc (2022) 54(9):2482–5. 10.1016/j.transproceed.2022.09.002

[83] Weber ML Ibrahim HN Lake JR. Renal Dysfunction in Liver Transplant Recipients: Evaluation of the Critical Issues. Liver Transpl (2012) 18(11):1290–301. 10.1002/lt.23522 22847917

[84] Solé A Zurbano F Borro JM Monforte V Ussetti P Santos F. Prevalence and Diagnosis of Chronic Kidney Disease in Maintenance Lung Transplant Patients: ICEBERG Study. Transpl Proc (2015) 47(6):1966–71. 10.1016/j.transproceed.2015.04.097 26293082

[85] Hernando C Hernando C Panizo N Collado-Boira E Folch-Ayora A Martínez-Navarro I Renal Function Recovery Strategies Following Marathon in Amateur Runners. Front Physiol (2022) 13:812237. 10.3389/fphys.2022.812237 35295572 PMC8918951

[86] Mansour SG Martin TG Obeid W Pata RW Myrick KM Kukova L The Role of Volume Regulation and Thermoregulation in AKI During Marathon Running. Clin J Am Soc Nephrol (2019) 14(9):1297–305. 10.2215/CJN.01400219 31413064 PMC6730516

[87] Kim J Lee J Kim S Ryu HY Cha KS Sung DJ. Exercise-Induced Rhabdomyolysis Mechanisms and Prevention: A Literature Review. J Sport Health Sci (2016) 5(3):324–33. 10.1016/j.jshs.2015.01.012 30356493 PMC6188610

[88] Chapman CL Johnson BD Parker MD Hostler D Pryor RR Schlader Z. Kidney Physiology and Pathophysiology During Heat Stress and the Modification by Exercise, Dehydration, Heat Acclimation and Aging. Temperature (2020) 8(2):108–59. 10.1080/23328940.2020.1826841 PMC8098077 33997113

[89] Juett LA James LJ Mears SA. Effects of Exercise on Acute Kidney Injury Biomarkers and the Potential Influence of Fluid Intake. Ann Nutr Metab (2020) 76(Suppl. 1):53–9. 10.1159/000515022 33774615

[90] Rizzo L Thompson MW. Cardiovascular Adjustments to Heat Stress During Prolonged Exercise. The J Sports Med Phys Fitness (2018) 58(5):727–43. 10.23736/S0022-4707.17.06831-1 28181776

[91] Rojas-Valverde D Martínez-Guardado I Sánchez-Ureña B Timón R Scheer V Pino-Ortega J Outpatient Assessment of Mechanical Load, Heat Strain and Dehydration as Causes of Transitional Acute Kidney Injury in Endurance Trail Runners. Int J Environ Res Public Health (2021) 18(19):10217. 10.3390/ijerph181910217 34639516 PMC8508486

[92] Li Z McKenna Z Fennel Z Nava RC Wells A Ducharme J The Combined Effects of Exercise-Induced Muscle Damage and Heat Stress on Acute Kidney Stress and Heat Strain During Subsequent Endurance Exercise. Eur J Appl Physiol (2022) 122(5):1239–48. 10.1007/s00421-022-04919-1 35237867

[93] Houck J McKenna Z Fennel Z Ducharme J Wells A Mermier C The Effect of Interval and Continuous Work on Markers of Acute Kidney Injury in a Hot Environment. Eur J Appl Physiol (2022) 122(11):2437–50. 10.1007/s00421-022-05030-1 35999474

[94] Luks AM Swenson ER Bärtsch P. Acute High-Altitude Sickness. Eur Respir Rev (2017) 26(143):160096. 10.1183/16000617.0096-2016 28143879 PMC9488514

[95] Davies AJ Kalson NS Stokes S Earl MD Whitehead AG Frost H Determinants of Summiting Success and Acute Mountain Sickness on Mt Kilimanjaro (5895 M). Wilderness Environ Med (2009) 20(4):311–7. 10.1580/1080-6032-020.004.0311 20030437

[96] Gieszer B Radeczky P Farkas A Csende K Mészáros L Török K Lung Transplant Patients on Kilimanjaro. Transpl Proc (2019) 51(4):1258–62. 10.1016/j.transproceed.2019.04.004 31101210

[97] Weber U Schiefer J Mühlbacher J Bernardi MH Ortner CM Jaksch P. High Altitude Trekking After Lung Transplantation: A Prospective Study Using Lung Ultrasound to Detect Comets Tails for Interstitial Pulmonary Edema in Lung Transplant Recipients and Healthy Volunteers. Transpl Int (2018) 31(11):1245–53. 10.1111/tri.13307 29928768

[98] Pirenne J Van Gelder F Kharkevitch T Nevens F Verslype C Peetermans WE Tolerance of Liver Transplant Patients to Strenuous Physical Activity in High-Altitude. Am J Transpl (2004) 4(4):554–60. 10.1111/j.1600-6143.2004.00363.x 15023147

[99] Wang B Li ZL Zhang YL Wen Y Gao YM Liu BC. Hypoxia and Chronic Kidney Disease. EBioMedicine (2022) 77:103942. 10.1016/j.ebiom.2022.103942 35290825 PMC8921539

[100] Wang SY Gao J Zhao JH. Effects of High Altitude on Renal Physiology and Kidney Diseases. Front Physiol (2022) 13:969456. 10.3389/fphys.2022.969456 36338473 PMC9630589

[101] Wang H Tang C Dang ZH Yong A Liu L Wang S Clinicopathological Characteristics of High-Altitude Polycythemia-Related Kidney Disease in Tibetan Inhabitants. Kidney Int (2022) 102:196–206. 10.1016/j.kint.2022.03.027 35513124

[102] Kim SR Choi S Kim K Chang J Kim SM Cho Y Association of the Combined Effects of Air Pollution and Changes in Physical Activity With Cardiovascular Disease in Young Adults. Eur Heart J (2021) 42(25):2487–97. 10.1093/eurheartj/ehab139 33780974

[103] Krittanawong C Qadeer YK Lavie CJ. Air Pollution, Physical Activity, and Lifespan. Mayo Clinic Proc (2023) 98(8):1113–5. 10.1016/j.mayocp.2023.06.009 37536798

[104] Hallett AM Feng Y Jones MR Bush EL Merlo CA Segev DL Ambient Air Pollution and Adverse Waitlist Events Among Lung Transplant Candidates. Transplantation (2022) 106(5):1071–7. 10.1097/TP.0000000000003837 34049363 PMC8613310

[105] Benmerad M Slama R Botturi K Claustre J Roux A Sage E Chronic Effects of Air Pollution on Lung Function After Lung Transplantation in the Systems Prediction of Chronic Lung Allograft Dysfunction (SysCLAD) Study. Eur Respir J (2017) 49(1):1600206. 10.1183/13993003.00206-2016 28100545

[106] Lauritzen P Mcclure M Smith ML Trew A. The Gift of Life and the Common Good. The Need for a Communal Approach to Organ Procurement. Hastings Cent Rep (2001) 31(1):29–35. 10.2307/3528731 11478091

[107] Siminoff LA Chillag K. The Fallacy of the "Gift of Life. Hastings Cent Rep (1999) 29(6):34–41. 10.2307/3527870 10641243

[108] Doi S Sekiguchi M Motoya R Kanazawa J Sato M Harada H Associations Between Recipients' Feelings of Guilt for Donor and Depressive Symptoms Before Living Kidney Transplantation. Transplant Proc (2022) 54(3):622–6. 10.1016/j.transproceed.2021.10.033 35307190

[109] Didier S Vauthier JC Gambier N Renaud P Chenuel B Poussel M. Substance Use and Misuse in a Mountain Ultramarathon: New Insight Into Ultrarunners Population? Res Sports Med (2017) 25(2):244–51. 10.1080/15438627.2017.1282356 28114830

[110] Martínez S Aguiló A Moreno C Lozano L Tauler P. Use of Non-Steroidal Anti-Inflammatory Drugs Among Participants in a Mountain Ultramarathon Event. Sports (2017) 5(1):11. 10.3390/sports5010011 29910371 PMC5969007

[111] Rotunno A Schwellnus MP Swanevelder S Jordaan E Janse Van Rensburg DC Derman W. Novel Factors Associated With Analgesic and Anti-Inflammatory Medication Use in Distance Runners: Pre-Race Screening Among 76 654 Race Entrants-SAFER Study VI. Clin J Sport Med (2018) 28(5):427–34. 10.1097/JSM.0000000000000619 29944515

[112] Harirforoosh S Asghar W Jamali F. Adverse Effects of Nonsteroidal Antiinflammatory Drugs: An Update of Gastrointestinal, Cardiovascular and Renal Complications. J Pharm Pharm Sci (2013) 16(5):821–47. 10.18433/j3vw2f 24393558

[113] Bhagat V Pandit RA Ambapurkar S Sengar M Kulkarni AP. Drug Interactions Between Antimicrobial and Immunosuppressive Agents in Solid Organ Transplant Recipients. Indian J Crit Care Med (2021) 25(1):67–76. 10.5005/jp-journals-10071-23439 33603305 PMC7874296

[114] Pilch NA Sell ML McGhee W Venkataramanan R. Important Considerations for Drugs, Nutritional, and Herbal Supplements in Pediatric Solid Organ Transplant Recipients. Pediatr Transpl (2021) 25(1):e13881. 10.1111/petr.13881 33142023

[115] Sheiner PA Mor E Chodoff L Glabman S Emre S Schwartz ME Acute Renal Failure Associated With the Use of Ibuprofen in Two Liver Transplant Recipients on FK506. Transplantation (1994) 57(7):1132–3. 10.1097/00007890-199404000-00026 7513099

[116] Garthe I Maughan RJ. Athletes and Supplements: Prevalence and Perspectives. Int J Sport Nutr Exerc Metab (2018) 28(2):126–38. 10.1123/ijsnem.2017-0429 29580114

[117] Mathews NM. Prohibited Contaminants in Dietary Supplements. Sports Health (2018) 10(1):19–30. 10.1177/1941738117727736 28850291 PMC5753965

[118] Daher J Mallick M El Khoury D. Prevalence of Dietary Supplement Use Among Athletes Worldwide: A Scoping Review. Nutrients (2022) 14(19):4109. 10.3390/nu14194109 36235761 PMC9570738

[119] Foroncewicz B Mucha K Gryszkiewicz J Florczak M Mulka M Chmura A Dietary Supplements and Herbal Preparations in Renal and Liver Transplant Recipients. Transpl Proc (2011) 43(8):2935–7. 10.1016/j.transproceed.2011.08.008 21996193

[120] Burke LM Castell LM Casa DJ Close GL Costa RJS Desbrow B International Association of Athletics Federations Consensus Statement 2019: Nutrition for Athletics. Int J Sport Nutr Exerc Metab (2019) 29(2):73–84. 10.1123/ijsnem.2019-0065 30952204

[121] Nolte Fong JV Moore LW. Nutrition Trends in Kidney Transplant Recipients: The Importance of Dietary Monitoring and Need for Evidence-Based Recommendations. Front Med (2018) 5:302. 10.3389/fmed.2018.00302 PMC6220714 30430111

[122] Chadban S Chan M Fry K Patwardhan A Ryan C Trevillian P The CARI Guidelines. Protein Requirement in Adult Kidney Transplant Recipients. Nephrology (2010) 15(Suppl. 1):S68–71. 10.1111/j.1440-1797.2010.01238.x 20591048

[123] Metzger M Yuan WL Haymann JP Flamant M Houillier P Thervet E Association of a Low-Protein Diet With Slower Progression of CKD. Kidney Int Rep (2017) 3(1):105–14. 10.1016/j.ekir.2017.08.010 29340320 PMC5762958

[124] Hahn D Hodson EM Fouque D. Low Protein Diets for Non-Diabetic Adults With Chronic Kidney Disease. Cochrane Database Syst Rev (2020) 10(10):CD001892. 10.1002/14651858.CD001892.pub5 33118160 PMC8095031

[125] Tesch PA. Exercise Performance and Beta-Blockade. Sports Med (2012) 2(6):389–412. 10.2165/00007256-198502060-00002 2866577

[126] Bonet S Agustí A Arnau JM Vidal X Diogène E Galve E Beta-Adrenergic Blocking Agents in Heart Failure: Benefits of Vasodilating and Non-Vasodilating Agents According to Patients' Characteristics: A Meta-Analysis of Clinical Trials. Arch Intern Med (2000) 160(5):621–7. 10.1001/archinte.160.5.621 10724047

[127] Winkelmayer WC Kewalramani R Rutstein M Gabardi S Vonvisger T Chandraker A. Pharmacoepidemiology of Anemia in Kidney Transplant Recipients. J Am Soc Nephrol (2004) 15(5):1347–52. 10.1097/01.asn.0000125551.59739.2e 15100376

[128] Janaudis-Ferreira T Tansey CM Mathur S Blydt-Hansen T Lamoureaux J Räkel A The Effects of Exercise Training in Adult Solid Organ Transplant Recipients: A Systematic Review and Meta-Analysis. Transpl Int (2021) 34(5):801–24. 10.1111/tri.13848 33608971

[129] Wallen MP Skinner TL Pavey TG Hall A Macdonald GA Coombes JS. Safety, Adherence and Efficacy of Exercise Training in Solid-Organ Transplant Candidates: A Systematic Review. Transpl Rev (2016) 30(4):218–26. 10.1016/j.trre.2016.07.004 27496067

[130] Qureshi R Mayo-Wilson E Li T. Harms in Systematic Reviews Paper 1: An Introduction to Research on Harms. J Clin Epidemiol (2022) 143:186–96. 10.1016/j.jclinepi.2021.10.023 34742788 PMC9126149

[131] Qureshi R Mayo-Wilson E Rittiphairoj T McAdams-DeMarco M Guallar E Li T. Harms in Systematic Reviews Paper 2: Methods Used to Assess Harms Are Neglected in Systematic Reviews of Gabapentin. J Clin Epidemiol (2022) 143:212–23. 10.1016/j.jclinepi.2021.10.024 34742789 PMC9875742

[132] Beedie CJ Foad AJ. The Placebo Effect in Sports Performance: A Brief Review. Sports Med (2009) 39(4):313–29. 10.2165/00007256-200939040-00004 19317519

[133] Rolid K Andreassen AK Yardley M Gude E Bjørkelund E Authen AR Long-Term Effects of High-Intensity Training vs Moderate Intensity Training in Heart Transplant Recipients: A 3-Year Follow-Up Study of the Randomized-Controlled HITTS Study. Am J Transpl (2020) 20(12):3538–49. 10.1111/ajt.16087 32484261

[134] Klaassen G Zelle DM Navis GJ Dijkema D Bemelman FJ Bakker SJL Lifestyle Intervention to Improve Quality of Life and Prevent Weight Gain After Renal Transplantation: Design of the Active Care After Transplantation (ACT) Randomized Controlled Trial. BMC Nephrol (2017) 18(1):296. 10.1186/s12882-017-0709-0 28915863 PMC5599936

[135] Cappelle M Masschelein E Vos R Van Remoortel H Smets S Vanbekbergen J High-Intensity Training for 6 Months Safely, But Only Temporarily, Improves Exercise Capacity in Selected Solid Organ Transplant Recipients. Transpl Proc (2021) 53(6):1836–45. 10.1016/j.transproceed.2021.03.040 34049699

[136] Dagner V Clausson EK Jakobsson L. Prescribed Physical Activity Maintenance Following Exercise Based Cardiac Rehabilitation: Factors Predicting Low Physical Activity. Eur J Cardiovasc Nurs (2019) 18(1):21–7. 10.1177/1474515118783936 29905494

[137] Beauchamp MK Evans R Janaudis-Ferreira T Goldstein RS Brooks D. Systematic Review of Supervised Exercise Programs After Pulmonary Rehabilitation in Individuals With COPD. Chest (2013) 144(4):1124–33. 10.1378/chest.12-2421 23429931

[138] Malaguti C Dal Corso S Janjua S Holland AE. Supervised Maintenance Programmes Following Pulmonary Rehabilitation Compared to Usual Care for Chronic Obstructive Pulmonary Disease. Cochrane Database Syst Rev (2021) 8(8):CD013569. 10.1002/14651858.CD013569.pub2 34404111 PMC8407510

[139] Leunis S Vandecruys M Cornelissen V Van Craenenbroeck AH De Geest S Monbaliu D Physical Activity Behaviour in Solid Organ Transplant Recipients: Proposal of Theory-Driven Physical Activity Interventions. Kidney Dial (2022) 2(2):298–329. 10.3390/kidneydial2020029

[140] De Geest S Zúñiga F Brunkert T Deschodt M Zullig LL Wyss K Powering Swiss Health Care for the Future: Implementation Science to Bridge "The Valley of Death. Swiss Med Wkly (2020) 150:w20323. 10.4414/smw.2020.20323 33022070

